# Supplementary material for: Tissue block staining and domestic adhesive tape yield qualified integral sections of adult mouse orbits and eyeballs
Source: PLoS One. 2021 Aug 4;16(8):e0255363. doi: 10.1371/journal.pone.0255363 (PMC8336840; doi:10.1371/journal.pone.0255363)
Supplement: S1 File — Complementary information of step-by-step protocol of BTA (Block staining, tape aided sectioning, and aqueously mounting), applications of BTA in the tissues rather than orbits, and original observational data. (DOCX) [file pone.0255363.s001.docx]

Supporting information

**Tissue block staining and domestic adhesive tape yield qualified integral sections of adult mouse orbits or eyeballs**

Zhongmin Li^1*^, Martin Ungerer^2^, Julia Faßbender^1^, Clara Wenhart^1^, Hans-Peter Holthoff^1^, Goetz Muench^1^

^1^Advancecor GmbH, 82152 Martinsried, Germany.

^2^ISAR Bioscience Institute, 82152 Planegg, Germany.

*, Correspondence and requests for materials should be addressed to Z. L. (email: [li@advancecor.com](mailto:li@advancecor.com))

1. Step-by-step protocol of BTA - “Block staining, tape aided sectioning, and aqueously mounting”.

**S1 Fig. Preparation of aluminum foil mold.**

A. Materials required for the mold. The strip of the foil is rolled into a syringe shaft of the diameter needed and fastened with a piece of adhesive tape (B). Cares are taken to ensure a plane surface in one end of the foil cylinder. The cylinder roll is pulled down and the aluminum foil mold is ready for use (C).

**S2 Fig. Embedding.**

A. A slide is placed on a metal block, which had been frozen in dry ice beforehand. B. One drop of OCT (optimal cutting temperature medium) is put on the slide and the oriented specimen on the OCT immediately after. C. An aluminum foil roll (mold) is sheathed onto the specimen and filled with OCT. The frozen tissue block is ready for sectioning.

**S3 Fig. Preparation of slips of adhesive tape.**

A. A stripe of aluminum foil with 2 mm in width is anchored onto a cutting pad with adhesive tape on both ends. B. Paste the adhesive tape (Tesa) on the pad and overlay the stripe with the tape edge in line with the upper border of the aluminum stripe. C. Cross incisions (indicated by green dotted lines) with 15 mm apart are made. Peel off the slip of tape with fine tweezers secured on the handle for tape-assisted sectioning.

**S4 Fig. Adhesive tape-aided sectioning.**

A. Mount a tissue block onto the cryostat chuck using OCT and trim the block. B. Peel a slip of tape off the cutting pad with fine tweezers secured on the handle and press the adhesive side of the tape onto the cutting surface of the sample block. Ensure that the cutting surface of the sample is entirely covered by the tape. Section the sample in one slow, but a continuous movement. C. Use forceps to lift the freshly sectioned tissue by handle on the tape and secure the section onto the glass slide by double adhesive tape (optional step for frozen store).

**S5 Fig. Coverslipping.**

A. Dip a tape-section into 20% glycerol, clip the handle off, and place the tape-section on a slide with the tissue section up. B. Mount one coverslip on the section. Note: freshly-prepared 20% glycerol should be balanced at room temperature for at least 30 min before application.

1. Applications of BTA in the tissues rather than orbits.

It is also rather challenging to get integral and undamaged staining sections of joints and brains in traditional histological processing, especially for thin sections. With the new protocol of BTA developed, we tested the feasibility of applications in the tissues.

 For the preparation of the joint tissue blocks, one 20 weeks old male DBA/1 mouse (Janvier Labs, France) was euthanized. The right hind paws were amputated at the level immediately above the external malleolus. The skin was peeled off and the skinless paws were fixed in 4% paraformaldehyde at 4°C overnight. After 3 weeks of decalcification and tissue block staining, tape-aided 5 µm thick sagittal staining section was acquired in the same way as the protocol of BTA described. The results are shown as in the images of S6 Fig.

 One ten weeks` old male C57BL/6J mouse weighing 25 grams was prepared for the brain and one nine weeks’ old male C57BL/6J mouse weighing 23 grams for the other tissues including heart, lung, kidney, spleen, and liver. Those animals were euthanized and the tissues were dissected. After 24 h fixation in 4% paraformaldehyde, the tissue blocks went through block staining, embedding, tape-aided 5 µm thick coronary sectioning, and aqueous mounting with the protocol of BTA as described above. The images acquired for the brain are displayed in S7 Fig. The results for the other tissues are shown as in the images of S8 & 9 Figs.

**S6 Fig. Images of the right hind paw from one 20 week-old male DBA/1 mouse.**

The paw was processed with the protocol of BTA after skinning, fixation, and decalcification. The lower magnification image of the middle sagittal staining section (HE) is shown in (A). In each tarsal joint, articular cartilage, capsule, cavity and synovia were easily distinguished in (B) and (C). (B) and (C) are local magnifications of (A).

**S7 Fig. Coronary cerebellum sections from a 10 week-old C57BL/6J mouse.**

The protocol of BTA was employed in the tissue processing. The lower magnification images of (A) show that the brain was stained uniformly. The morphology of cellular nuclei was clear (D–E). The images (B) and (C) are local magnifications of (A); and images of (D) and (E) are the local magnifications of (B) and (C) respectively.

**S8 Fig. Images of a heart and kidney from a 9 week-old C57BL/6 mouse.**

**
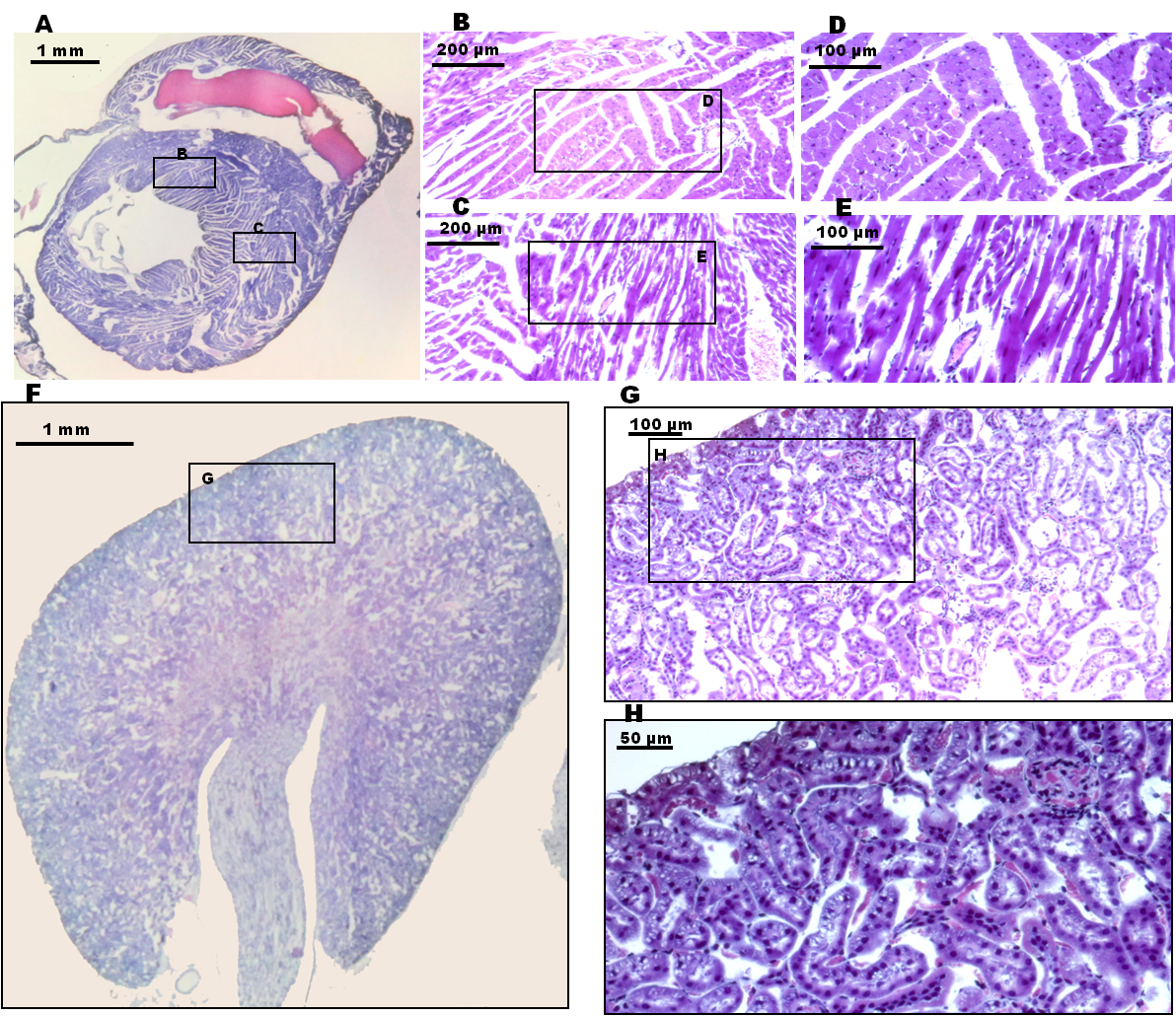
**

HE staining was performed with the protocol of BTA. The lower magnification images of the mouse heart in (A) and the kidney in (F) display uniformed staining. The morphology of cellular nuclei was clear in the higher magnification images (D, E, and H). The images (B) and (C) are local magnifications of (A), and the image (G) the local magnification of (F). The further magnification images (D, E and H) are of the locals of images (B, C and G) respectively.

**S9 Fig. Images of a lung and spleen.**

The lung and the spleen were derived from a 9 week-old C57BL/6 mouse. HE staining was performed with the BTA protocol. The lower magnification images of the mouse lung in (A) and the spleen in (D) display uniformed staining. The morphology of cellular nuclei was clear in the higher magnification images (C and F). The images (B) and (E) are local magnifications of (A) and (D) respectively, and the images (C) and (F) are local magnifications of (B) and (E).

| 3. Original observational data.  **S10 Table. The results of observation on orbital sections.** | | | | | | | | | | |  |  |  |  |  |  |
| --- | --- | --- | --- | --- | --- | --- | --- | --- | --- | --- | --- | --- | --- | --- | --- | --- |
| methods | animals | section 1 | | | section 2 | | | **section 3 (best one of 10)** | | | section 4 | | | section 5 | | |
|  |  | mac | x 2.5 | x 10 | mac | x 2.5 | x 10 | **mac** | **x 2.5** | **x 10** | mac | x 2.5 | x 10 | mac | x 2.5 | x 10 |
| BTA | 1 | a2 | - | - | a1 | - | - | **-** | **-** | **a9** | - | - | a9 | - | - | a9 |
|  | 2 | a1,a4 | - | - | a4 | a8 | - | **-** | **-** | **a11** | - | - | - | - | - | a11 |
|  | 3 | - | - | - | a1 | a7 | - | **-** | **-** | **-** | - | - | - | - | - | - |
| BTO | 1 | a2 | a7 | a12 | a1 | a7 | a12 | **-** | **-** | **a10,a11** | a1 | - | a12 | a1 | a7 | a12 |
|  | 2 | a1 | a7 | a10 | a1 | a7 | a10 | **a1** | **a7** | **a12** | a1 | a7 | a10 | a1 | a7 | a10 |
|  | 3 | a1 | - | a12 | a1 | a8 | a12 | **a1,a3** | **a7** | **a10** | a1 | a8 | - | a1 | - | a12 |
| BWA | 1 | a3,a4 | a7,a8 | a12 | a3,a4 | a7,a8 | a12 | **a1,a3,a4** | **a8** | **a12** | a3,a4 | a7,a8 | - | a3,a4 | a7,a8 | a12 |
|  | 2 | a1,a3,a4 | a7,a8 | a11,a12 | a1,a3,a4 | a7,a8 | a11,a12 | **a3,a4** | **a7,a8** | **a12** | a1,a3,a4 | a7,a8 | a11,a12 | a1,a3,a4 | a7,a8 | a11,a12 |
|  | 3 | a1,a4 | a6,a7,a8 | a12 | a1,a4 | a6,a7,a8 | a12 | **a1,a3,a4** | **a7,a8** | **a11,a12** | a1,a4 | a6,a7,a8 | a12 | a1,a4 | a6,a7,a8 | a12 |
| BWO | 1 | a1,a3,a4 | a7,a8 | a12 | a1,a3,a4 | a7,a8 | a12 | **a1,a4** | **a6,a7,a8** | **a12** | a1,a3,a4 | a7,a8 | a12 | a1,a3,a4 | a7,a8 | a12 |
|  | 2 | a1,a3,a4 | a7,a8 | - | a1,a3,a4 | a7,a8 | - | **a1,a3,a4** | **a7,a8** | **a12** | a1,a3,a4 | a7,a8 | - | a1,a3,a4 | a7,a8 | - |
|  | 3 | a1 | - | a10,a12 | a1 | - | a12 | **a1,a3,a4** | **a7,a8** | **-** | a1 | - | a10,a12 | a1 | - | a10,a12 |
| TSA | 1 | a1 | - | a10,a12 | a1 | - | a12 | **a1** | **-** | **a10,a12** | a1 | - | a10,a12 | a1 | - | a10,a12 |
|  | 2 | - | a8 | a12 | a4 | a8 | a11,a12 | **a4** | **a8** | **a12** | a4 | a8 | a12 | a4 | a8 | a12 |
|  | 3 | a1,a4 | a7 | - | a1,a4 | a7 | a12 | **a1,a4** | **a7** | **a12** | a1,a4 | - | a12 | a1,a4 | a7 | a12 |
| TSO | 1 | a4 | a6,a7 | a12 | a4 | a6,a7 | a12 | **a4** | **a6,a7** | **a12** | a4 | a6,a7 | a12 | a1 | a6,a7 | a12 |
|  | 2 | a3,a4 | - | a12 | a3,a4 | - | a12 | **a3,a4** | **a8** | **a12** | a3,a4 | - | a12 | a1 | a8 | a10 |
|  | 3 | a1,a3,a4 | a8 | a12 | a1 | a8 | a12 | **a1,a3,a4** | **a8** | **a12** | a1,a3,a4 | a8 | a12 | a3,a4 | a8 | a12 |
| WSA | 1 | a1 | a7,a8 | a11,a12 | a1 | a7,a8 | a11,a12 | **a1** | **a7,a8** | **a11,a12** | a1,a4 | a7,a8 | a11,a12 | a1,a3,a4 | a7,a8 | a12 |
|  | 2 | a1,a4 | a7,a8 | a12 | a4 | a7,a8 | a10,a12 | **a1,a4** | **a7,a8** | **a10,a12** | a1,a4 | a7,a8 | a10,a12 | a1,a4 | a7,a8 | a10,a12 |
|  | 3 | a1 | a8 | a10,a12 | a1 | a8 | a10,a12 | **a1** | **a8** | **a10,a12** | a1 | a8 | a10,a12 | a1 | a8 | a10,a12 |
| WSO | 1 | / | / | / | / | / | / | **a1,a4** | **a7,a8** | **a11,a12** | / | / | / | / | / | / |
|  | 2 | / | / | / | / | / | / | **a1,a4** | **a8** | **a12** | / | / | / | / | / | / |
|  | 3 | / | / | / | / | / | / | **a1,a4** | **a7,a8** | **a12** | / | / | / | / | / | / |

The observational outcomes are from the sections collected at 5 Bregman positions mentioned and expressed as -, normal; a1, section wrinkle or fold; a2, bubble under section; a3, tissue fissure; a4, missing part of tissue; a5, contaminant adheres; a6, shattering; a7, local tissue dislocation; a8, distorted tissue structure; a9, blurring view; a10, poor staining color contrast; a11 indistinct tissue structure; and a12, unusual cell or tissue aggregation. / , missing due to technically unrelated problems. The data on the middle sections highlighted was used for comparison among different methods.
